# Making teamwork work: enhancing teamwork and assessment in higher education

**DOI:** 10.1002/2211-5463.13936

**Published:** 2024-11-17

**Authors:** Nigel Francis, Connie Pritchard, Zoe Prytherch, Stephen Rutherford

**Affiliations:** ^1^ School of Biosciences Cardiff University UK

**Keywords:** assessment, collaborative learning, group‐based assessment, groupwork, team‐based assessment, teamwork

## Abstract

The ability to work in teams is one of the most sought‐after graduate skills by employers. However, team‐based learning activities, and especially team‐based assessments, are commonly disliked (even actively avoided) by students. Team‐based assessments are often problematic for students, mostly due to logistical problems and interpersonal difficulties. These difficulties often lead to dissatisfaction with the process and poor satisfaction responses in quality assessments of their teaching. This review takes a four‐way approach to evaluate current approaches to team assessment aimed at enhancing student engagement, satisfaction and learning gain. Firstly, we identify why team‐based activity is so important to include in our overall pedagogy in Higher Education. Secondly, we examine evidence from the literature on students' reactions to team‐based activities (especially focusing on assessment) and the reasons for both positive and negative perceptions. The third focus is on identifying the root of the problem from a pedagogic perspective and highlighting the deficiencies in approaches to team‐based activities that might lead to negative student perceptions. Finally, we discuss examples from the literature of where team‐based learning and assessment activities have been successful. Approaches to team‐based activities need to be more proactive and supportive so that students understand the dynamics of teams, how to plan team‐based activities, and how to deal with interpersonal issues positively and productively. Team‐based learning is arguably the least well‐taught element of our curricula, yet it is important and straightforward to address.

## Graphical abstract

Students in Higher Education are often resistant to team‐based learning activities and assessments. Student perceptions of positive impacts (learning gains, reduced assessment load and beneficial social interactions) are strongly outweighed by logistical challenges and perceptions of unfairness. Effective planning and support by educators can address these issues. We propose guidance to support effective and engaging team‐based learning tasks and assessments.

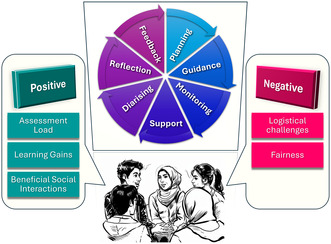

**Abbreviations**
AI: artificial intelligence
BARS: behaviourally anchored rating scales
CATME: comprehensive assessment of team member effectiveness

Teamwork is a cornerstone of modern educational practices, recognised for its potential to develop critical transferrable skills among students, such as collaborative working, communication, problem‐solving and leadership. This review focuses on the role of teamwork in higher education, where the approach is particularly valued for its ability to simulate working‐world professional environments where collaborative efforts are ubiquitous. Teamwork therefore not only enhances academic learning, but also prepares students for the workplace by developing these key graduate attributes [1, 2].

This review adopts the term ‘teamwork’ in preference to the alternative term for the pedagogy, ‘group work’. Teamwork is gaining in popularity as a term since it better reflects the discourse of the professional/graduate working environment to which this pedagogy relates. Referring to ‘teams’ rather than ‘groups’ may help reduce anxiety about working with others, especially among neurodiverse students, aligning with theories of compassionate pedagogy [3]. Team‐based and group‐based learning have subtle differences. ‘Team working’ is increasingly recognised as a fundamental competency in an increasingly more globalised, dynamic and complex work environment [4]. Zhang [5] defines the key differences between team‐ and group work as teamwork requiring a higher level of communication and coordination, which leads to a clearer understanding of the goals and expectations of team members. In contrast, group work may implicitly lack these depths of interaction and structure [6]. These distinctions highlight that teamwork is more structured and focused on collaborative interpersonal interactions, whereas group work may imply more task‐oriented with less emphasis on interpersonal issues [5]. Watson *et al*. [7] and De Prada *et al*. [4] highlight that teamwork involves a collection of different skills, behaviours and attitudes that must be taught if they are to become beneficial to students outside of the teamwork and/or educational setting.

Despite its many benefits, implementing teamwork tasks is full of challenges, especially for the modern student [8]. Issues such as uneven participation, free riding/loading and conflicts among team members can hinder the effectiveness of team projects and lead to student dissatisfaction [1]. Additionally, there may be external logistical challenges for students to engage with team‐based tasks, such as part‐time employment or caring responsibilities [9]. The success of team‐based tasks is heavily reliant on the educators' strategic planning and scaffolding of activities to proactively address these issues [5, 10], as well as scheduling space within the timetable for team‐based activities to be carried out [5, 9, 11].

The primary objective of this review is to synthesise existing strategies and best practices for effective teamwork and team assessment in higher education. The review aims to provide educators with comprehensive insights into how to maximise the benefits of teamwork while mitigating its challenges. This includes exploring structured approaches to team tasks, methods for enhancing student engagement proactively, the integration of technology, and effective assessment techniques to ensure fair and constructive evaluations.

## Theoretical framework

### Theoretical basis and benefits of teamwork

The theoretical underpinnings of teamwork align closely with those of collaborative learning, through involving students working together to achieve common academic goals. Collaborative learning is itself rooted in social constructivism and sociocultural models of learning, which propose that learning is a socially mediated process. Vygotsky's model of the ‘Zone of Proximal Development’ [12] emphasises the importance of social interactions in cognitive development, suggesting that students learn more effectively when collaborating with others (in Vygotsky's model, this is ideally a more experienced mentor). Mercer [13, 14] reimagined Vygotsky's model as the ‘Intermental Development Zone’, which focuses on the mutual learning gain achieved by both parties in a socially‐based learning interaction. Mercer focused particularly on peer‐to‐peer interactions and the power of peers undertaking communal problem‐solving. These perspectives are supported by the work of Mazur [15], who highlights the role of peer instruction in facilitating deeper understanding and retention of knowledge.

Collaborative learning offers numerous academic and social benefits [16], such as encouraging active learning, critical thinking, and the ability to apply knowledge in practical contexts [17, 18]. Socially, collaborative learning helps students develop essential interpersonal skills such as communication, negotiation, and conflict resolution [19, 20]. Collaborative learning can also facilitate the development of networks of support, such as ‘personal learning networks’ [21, 22]. Furthermore, working in diverse teams exposes students to different perspectives, fostering a more inclusive learning environment and preparing them for the collaborative nature of the modern workplace [4].

### Definition and types of teams

According to Davis [23], there are three primary types of teams commonly encountered in higher education:

1. *Informal teams* are *ad hoc* groupings used within a single class session to facilitate discussion or brief collaborative activities. Examples include ‘turn‐pair‐share’ exercises where students discuss a topic with a neighbour before sharing it with the larger cohort. Work by Smith *et al*. [24] provides an in‐depth analysis of peer grouping and attainment within lectures.
2. *Formal teams* are established to complete specific tasks, such as a research project, presentation or laboratory work, over a longer period. Formal teams are typically assigned by the educator and may last for the period of time of an assignment and/or the duration of a module/course. Lacey *et al*. [25] have shown that initial instructor‐selected groups can result in more dynamic future groupings in the laboratory setting.
3. *Study teams* are primarily student‐driven, self‐organised, and continuous over a long‐term period. Study teams form to provide mutual peer support in understanding course material and preparing for assessments, often lasting for the duration of a module or even over an entire course.

Employers in bioscience research and clinical settings commonly highlight the importance of team‐based experience [24]. Embedding teamwork activities, and in particular assessed team activities, is therefore essential in preparing students to be employable global citizens. However, despite their importance, students often dislike, mistrust or resist teamwork activities [1, 26].

## The problem—why doesn't teamwork work?

**Fig. 1:**
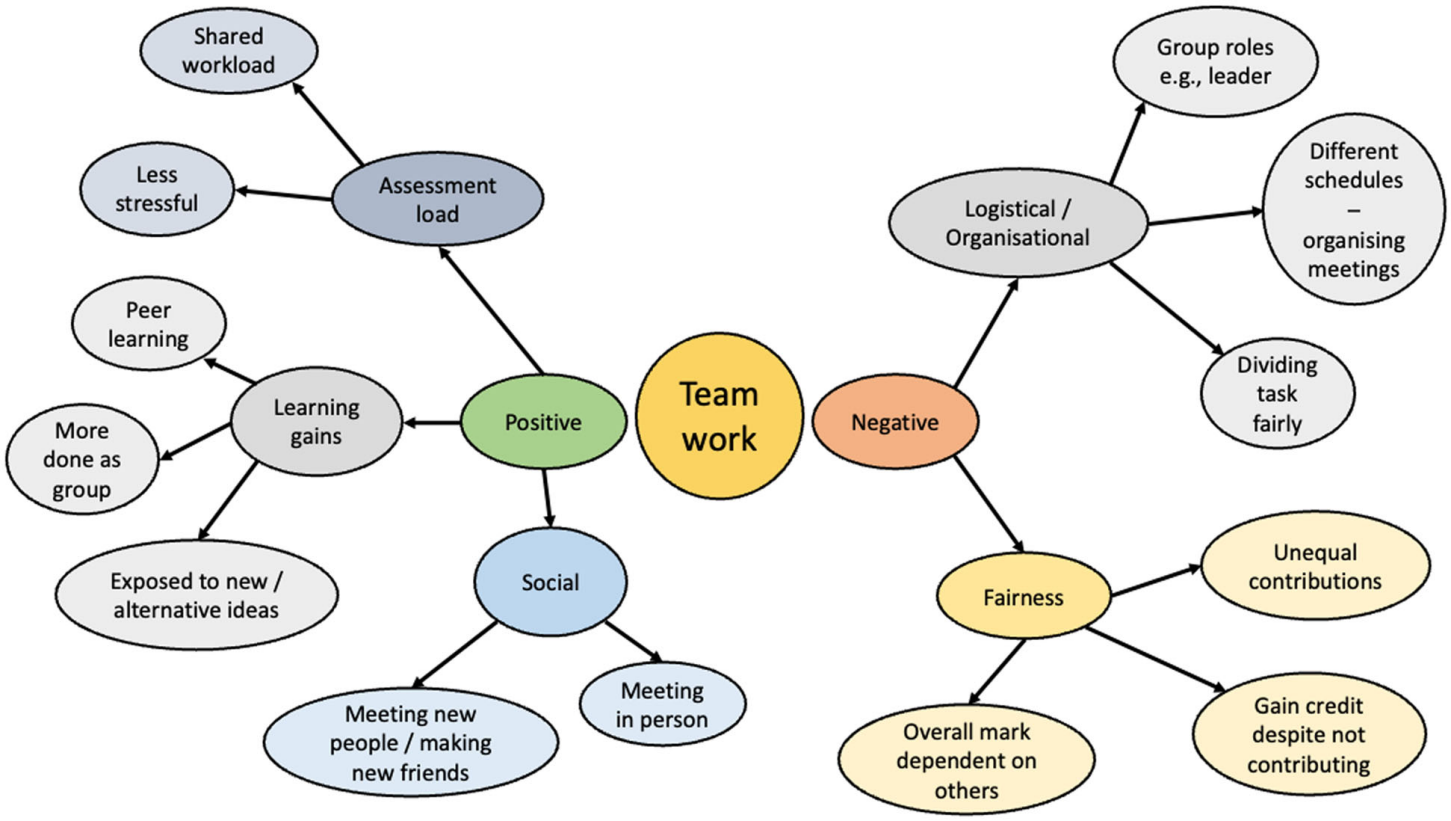
Thematic analysis of student perceptions of the benefits and challenges associated with teamwork. Factors identified by a preliminary analysis of student focus group responses [9] identifying students' perceptions of barriers and challenges to effective and engaging team‐based learning in Higher Education.

Teamwork assessments are infamous for the extent to which they are disliked and avoided by students [8]. Factors such as problems with group formation or interpersonal conflicts within a team, differential levels of engagement from team members, and the perceived unfairness of a student's reliance on another individual for their grade [1, 11, 26] make teamwork assignments potentially problematic. In addition, the rationale for making an assessment a team‐based activity is not always founded in sound pedagogic principles but rather can be a response to high student numbers and staff workload [1, 27]. These factors can contribute to poor student perceptions of teamwork assignments. Figure 1 highlights some issues raised by students from a qualitative study undertaken by Francis *et al*. [9] (in preparation).

One of the major reasons why team tasks, especially team assessments, fail or are limited in their impact is the general lack of scaffolding and support for students from academic staff in their use [1, 26]. In the authors' experience of teamwork reported across the sector, the most common team‐based assessment approach appears to be setting a team‐based task, dividing students into teams, and then receiving the output from the team activity. This approach implicitly assumes that students will know how to facilitate the development of the task and possess the interpersonal skills necessary for effective team working [1, 28]. It is rare for the process to be supported as an ongoing activity or for students to receive the appropriate training required for them to manage the teamwork activities effectively [29]. Therefore, a key element to success is to proactively address typical challenges for teamwork and use this activity as a learning experience for all involved. Socially shared regulation of teamwork tasks has been shown to encourage a more positive teamwork environment, leading to a more balanced contribution from team members and enhancing collaborative and cooperative learning [29].

**Fig. 2:**
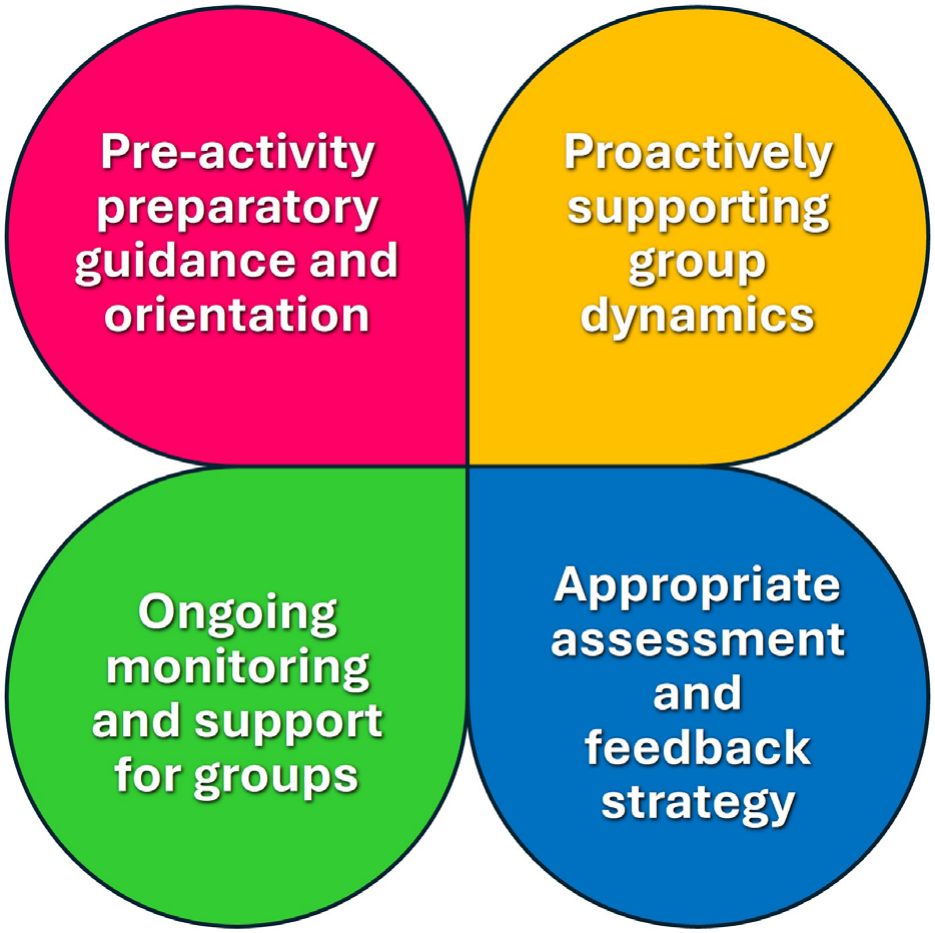
Four key elements to support effective teamwork activities. Based on the experience of the authors and guidance from the literature, we propose four key factors that will positively impact on team cohesion and success: Preparatory work and guidance before the start of the task; proactively supporting group dynamics through advice, training, planning and diarising the team activities; monitoring teams' progress and flagging interpersonal problems early; designing effective assessment tools, including peer evaluation of contribution, and effective feedback on outputs and team cohesion.

Above all, the key to success is in the effective scaffolding of the task and effective support of the teams as they undertake that task. Figure 2 summarises the four key factors that support success: Preparation of the students for teamwork through educating them in teamwork principles; encouraging the early division of roles and responsibilities to encourage positive group dynamics; scaffolding of the task and monitoring of the teams during the assignment, to identify any problems and address them; finally, ensuring effective assessment and feedback not only on the output but on the process and the participants in the team activity. The fundamental factors in these four areas are discussed in more detail below.

## Preparing students for teamwork: structured teamwork strategies

The first step to successful teamwork is forming teams themselves. This is discussed in more detail in Francis *et al*. [1], covering details of group sizes and approaches for forming groups. Opinion is divided between educators as to whether to assign teams, or to allow students to self‐select their own teams. There are contrasting benefits and challenges associated with both strategies [26, 30]. Self‐selected groups often avoid interpersonal conflicts but can be exclusory for minority demographics and neurodiverse students. Educator‐selected groups are more egalitarian and mirror the professional environment more closely, but they are more problematic to manage. One novel approach to group formation implemented by Sublett *et al*. [31] involved a ‘speed‐interviewing’ approach, allowing students to select groups based on shared interests and work ethics.

### Importance of clear roles and responsibilities

Implementing structured teamwork strategies that include assigning clear roles, effective division of labour and project planning can significantly enhance the effectiveness and fairness of teamwork. These logistical factors ensure that all students are actively kept engaged, accountable and contributing to the team's objectives [32]. A fundamental element in the success of teamwork is the establishment of clear roles and responsibilities. According to Steiner *et al*. [33], when students understand their specific roles within a team, it fosters accountability, helping ensure that each member contributes effectively to the collective goal. This clarity of purpose prevents the common problem of unequal participation, where some students may contribute significantly more than others. Clearly defined roles also help manage team dynamics by reducing confusion and potential conflicts about task ownership and responsibilities [33]. However, students are often unaware that it is acceptable to allocate different roles to different individuals according to their skill sets. The same applies to individual tasks within the larger assignment.

### Division of labour and task delegation

Effective division of labour and task delegation are critical components of structured teamwork, but this is one of the most common causes of dysfunction in team‐based activities. Sormunen *et al*. [32] highlight that dividing tasks among team members improves efficiency and enhances fairness. By allocating specific tasks to individuals based on their strengths and interests, teams can maximise productivity and ensure that all members are actively engaged. This strategy mimics practices in the graduate working environment [34]. Task allocation also allows students to take ownership of their assigned tasks, thereby increasing their motivation and commitment to the team's success [32]. Interestingly, a recent study at a Chinese university where teamwork was an unusual activity [35] highlighted that students adjusted their efforts based on teammates' contributions, reducing their own efforts when others contributed more and increasing efforts to compensate for less contributing members.

**Fig. 3:**
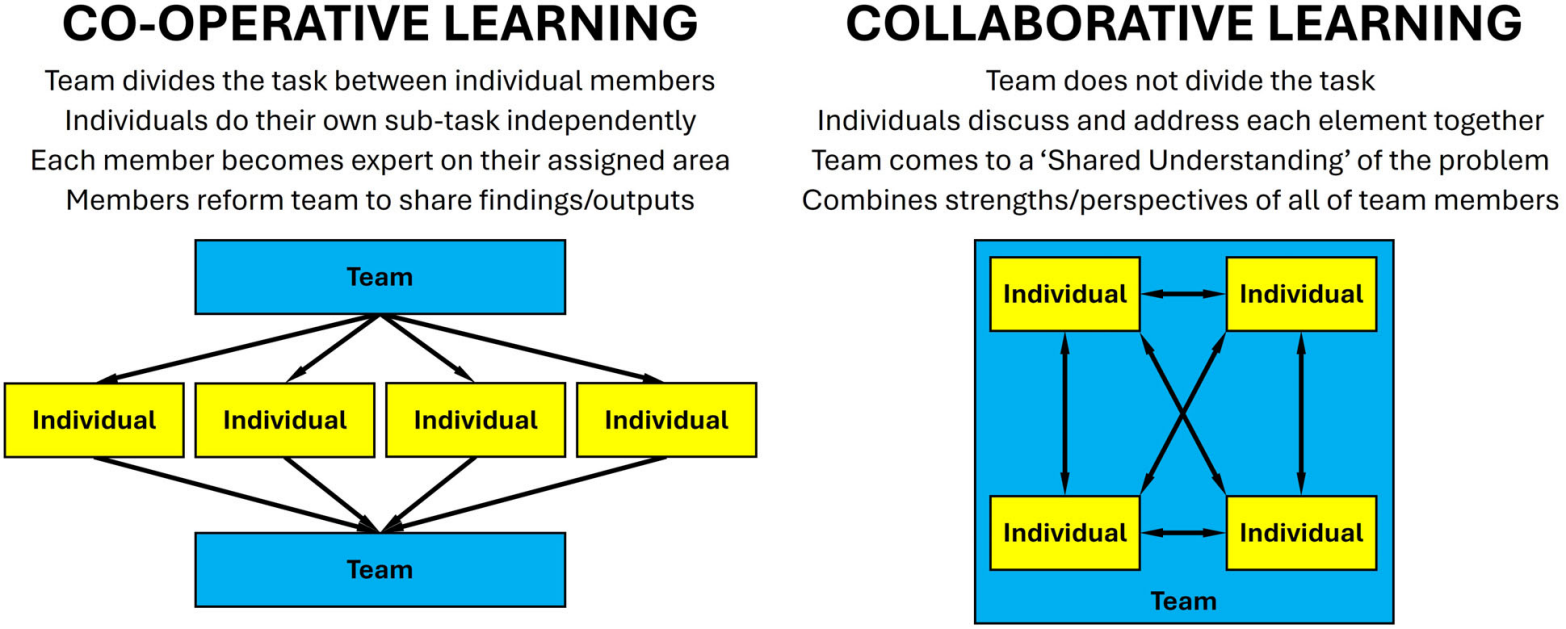
Comparison of Cooperative and Collaborative Learning. The relationship between individuals (yellow) and the Team (blue) is highlighted, as defined by Dillenbourg [36], with the arrows representing research/reporting lines. In cooperative learning, the team divides the task between the team members, who then work on their allocation individually, and then return to the team to group the individual outputs together. In Collaborative Learning, the team divides the task into sub‐tasks, but does not divide these sub‐tasks between individuals. Instead, the team as a whole works on each sub‐task, providing a collection of different perspectives on each sub‐task.

Team‐based activities can be described as either collaborative (working as a team on the same elements of a whole project) or cooperative (dividing the task for individuals to work on separate tasks) [36] (summarised in Fig. 3). Collaborative approaches are more likely to lead to effective team outcomes, as all partners in the team contribute. Cooperative activities, with their emphasis on dividing the task into discrete individual deliverables, may lead to more conflict if some team members do not deliver. Boud and Bearman [37] argue that collaborative learning should become the norm, with teamwork or peer learning becoming an expected part of the curriculum.

Collaborative and cooperative activities each have benefits and challenges, but regardless of the method, both strategies involve organising team activities so that each member's contribution is necessary for the team's overall success [5]. Understanding the underpinning principles for approaching team activities is essential for teams to function productively. Workshopping these approaches in a practical session is helpful for promoting active team participation and accountability. For example, using techniques like ‘jigsaw’ [38] can reveal the benefits of both collaborative and cooperative learning. In a ‘jigsaw’ activity, a task is broken down into components, each assigned to an individual in the team (a cooperative approach). Each individual then joins students from other teams who are addressing the same component, and these work as a new team on that one component (collaborative learning). Finally, the original team reforms and each student is responsible for learning and then teaching their new understanding of their component to their peers, ensuring everyone is involved and accountable not only for their own learning but for the group's as well. All team members then have an understanding of the whole project rather than an individual segment of the task [38], and each segment has been addressed collaboratively.

## Addressing interpersonal challenges in teams

Some major challenges to team‐based activities focus on interpersonal challenges within teams. Any team facing these challenges of differential contributions, interpersonal conflicts and communication may have a negative experience with a team‐based assessment [1, 9]. Addressing these issues proactively can have the positive impact of empowering students with the skills needed to resolve them [39].

### Conflict resolution and management

Conflict within teams is a common challenge that can disrupt collaborative efforts and negatively impact outcomes. Payne *et al*. [40] emphasise the importance of conflict resolution training for students, which can significantly enhance their ability to manage interpersonal issues constructively. Effective conflict resolution strategies include open communication, active listening and establishing clear team ground rules and procedures for addressing disputes [41]. Magana *et al*. [39] highlight that embedding conflict resolution training in coursework significantly enhances students' abilities to handle team disputes effectively, emphasising methods like active listening and setting clear communication norms. Similarly, Payne *et al*. [40] argue that establishing team ground rules proactively fosters an environment conducive to open dialogue and reduces conflicts. The educator plays a critical role in guiding this process by modelling good conflict resolution techniques and facilitating early interventions. This ensures that team interactions remain constructive and focused on collaborative success. Integrating these approaches can shift the educator's role from a passive overseer to an active facilitator, reinforcing students' academic and interpersonal growth.

### Free riding and social loafing

One of the most significant challenges in teamwork is where some team members contribute less effort than others, relying on their peers to carry the load, referred to variously (and interchangeably) as free riding, freeloading or social loafing. Macfarlane [42] highlights social loafing as a critical barrier to effective teamwork, often leading to student frustration and inequity. Noonan [27] further explains that freeloading can undermine the educational benefits of team activities, as it discourages full participation and can result in uneven learning experiences. A consequence of freeloading team members is the ‘sucker effect’, which is a counterintuitive reaction to free riding, whereby the more engaged students distance themselves from the task or team to avoid becoming the ‘sucker’ and doing all the work [43]. The sucker effect can be mitigated partially by encouraging more social interactions between team members, thereby reinforcing team dynamics and accountability [44].

### Strategies to ensure fair contribution

Proactive strategies can mitigate the issues of free‐riding and ensure fair contribution, such as:

1. Team contracts: establishing a contract at the task's outset can help set clear expectations for each team member's contributions [1]. This contract can include specific roles, deadlines, and consequences for non‐compliance, fostering a sense of accountability and commitment. The contract can be pre‐defined by the educator, but additional benefits may be gained from having the team members co‐create their own contract from a template, such as those provided on unihelper.io.
2. Peer evaluation of contributions: incorporating peer evaluation into the teamwork process allows students to review each other's contributions. Research shows that students are well‐placed to judge the contributions of their peers [45]. Peer evaluation of contribution to the task holds individuals accountable and provides valuable feedback that can improve team dynamics and performance. Anonymous peer evaluation methods, such as anonymous forms, can effectively ensure honesty and reduce bias. Online systems like WebPA (https://webpaproject.lboro.ac.uk) or Buddycheck (www.buddycheck.io) can help automate these processes [45, 46]. Encouraging teams to keep detailed notes of the group activities helps provide evidence to back up any claims of low‐ or non‐contributing team members.

When implemented effectively, these strategies can address common challenges in teamwork and promote a fairer and more productive collaborative learning environment. Interestingly, a recent study by Benning [47] revealed that from the student perspective, the method of group formation, group size, peer progress evaluations and a common grade are all important factors to reduce the likelihood of freeloading.

## Enhancing student engagement

### Active learning and working‐world applications

Engaging students in teamwork can be significantly enhanced through active learning strategies and the application of authentic, working‐world problems [7, 48]. Kriflik and Mullan [49] argue that actively teaching about team dynamics and collaborative skills greatly improves students' perceptions of teamwork tasks, leading to higher engagement levels. Similarly, Leight *et al*. [50] demonstrate that when students are involved in projects that mimic working‐world scenarios, their motivation and participation increase. These practical applications help students see the relevance of their work, thereby deepening their learning experience. The environment in which students carry out authentic team tasks can also be important to enhance student engagement with team tasks, for example, while on placements [2], or in a laboratory setting [11].

### Integrating relevant and dynamic tasks

Maintaining student interest in team‐based learning requires the integration of tasks that are both relevant and dynamic. Tasks should be designed to challenge students and require them to apply their knowledge creatively. This not only keeps students engaged but also helps develop critical thinking and problem‐solving skills. Incorporating a variety of task types, such as collaborative research projects, case studies and interactive simulations, can keep the work dynamic and engaging [1].

### Gamification to motivate and assess

Gamification, or the use of game‐like elements in non‐game contexts, can be a powerful tool for motivating students and enhancing teamwork. Moccozet *et al*. [51] found that integrating gamified elements, such as points and rewards, into team assessments not only increased student engagement but also provided a more accurate measure of individual contributions. Brar *et al*. [52] used a sci‐fi‐themed board game to demonstrate that student engagement with teamwork, their development of communication skills and self‐confidence can all be enhanced through gamified teaching resources. Emotional intelligence, life goal setting and motivation to study in peer and mixed groups have all been shown to be improved through a cooperative gamified approach [53]. By incorporating elements of competition and reward, gamification can make teamwork more appealing and encourage active participation from all members. Toda *et al*. [54] evaluated the effectiveness of such elements, showing that gamification in educational contexts, particularly teamwork, leverages the dynamics of cooperation, competition and social interaction to foster engagement, motivation, and collaboration. The authors state that the key to success is careful task design to balance competition and cooperation, ensuring both individual and team dynamics contribute positively to learning [54].

## Technological integration

### Use of collaborative technologies

The integration of collaborative technologies and online workspaces can significantly enhance the effectiveness of teamwork. Moccozet *et al*. [51] highlight that these tools facilitate better communication and coordination among team members, allowing for seamless collaboration regardless of physical location. Platforms such as Google Docs or Microsoft Teams provide shared spaces where students can work together in real time, share resources, and keep track of their progress [55].

### Tools for tracking and evaluating contributions

Various technological tools can be employed to ensure a fair and accurate assessment of individual contributions. Wikis, blogs and meeting logs are particularly useful in this regard. Wikis allow for collaborative document creation where every edit is tracked, making it easy to see who contributed to what [56]. Blogs can serve as reflective journals where students document their individual progress and contributions [57]. Meeting logs can record attendance and participation in team meetings, providing a clear record of each member's involvement [58]. These tools enhance transparency, and help identify and address any issues related to free‐riding or uneven participation [1, 56].

Team members have been shown to be well‐placed to judge the contribution of their peers. The use of automated online tools, such as WebPA [45] or Buddycheck [59], to adjust team marks based on peer and self‐evaluation can add an extra level of robustness to team‐based assessment and is appreciated by students. A key consideration of this approach is ensuring that the assessment is authentic [7, 50] and that both the peer and academic elements of the assessment are clearly visible and outlined to students before the commencement of the team project [60].

## Supporting teams during teamwork

An important element of effective team‐based learning is the support of the teams as they are undertaking the assignment [10, 61]. In teamwork assignments, there is often attention paid to the evaluation of contributions by team members, and approaches taken to resolve conflicts. However, many of the challenges arising from these issues can be forestalled by effective educator‐led support of teams during the teamwork process itself [62]. This support can be either interventionist or passive on the part of the educator.

### Setting goals and deliverables

A useful initial step is to guide teams in setting goals and key deliverables for the task and timetabling these deliverables, working backwards from the submission date [1]. A useful initial activity is to bring the teams together (an initial, facilitated, face‐to‐face meeting in a class is helpful for kick‐starting team cohesion), and guide them in goal setting, role allocation, and charting out a series of deliverables and landmark points in a timeline (see Francis *et al*. [1] for detailed suggestions of potential activities). Using a GANNT chart methodology is helpful here, as that highlights the multiple deliverables that a team‐based task typically involves. This session can also include the educator explaining the nuances of teamwork and suggesting potential self‐help solutions for team conflicts [63].

### Diarising the team's progress

It is useful to encourage (or require, as a part of the assessment) teams to keep a log of their activities [64]. This log can be a combination of a record of meetings, a contract between the team members, and a working space for research and development of the assignment output (adopted as a ‘Research Trail’ in an assessment case study by Rutherford and Prytherch [65]). The log can also potentially include a reflective element by the team on their approach. A log of activities also provides evidence of contribution levels to back up any issues in the peer evaluation of input. It can also serve as a passive means of identifying potential team problems on the part of the educator.

Collaborative documents are helpful here, such as the use of Google Docs, or shared documents on a platform such as Microsoft Teams. Periodic check‐ins by the educator enable them to track any disengagement of team members, and to flag lack of progress if the group is falling behind. Making this activity a small part of the grade for the team task is a good way of ensuring that students engage with this activity.

### Educator check‐ins

Proactive educator support is a means of proactively stalling team problems. Scheduling one or two short ‘check‐in’ meetings with the teams is an effective means of future‐proofing against any negative issues. Short (15‐min) meetings once or twice during a long‐term task need not be onerous on either the educator or the team members but can be extremely effective in highlighting any group issues, non‐engagement, or potential misunderstandings. Online conferencing platforms can be a means of managing these meetings, so they are logistically straightforward to hold and non‐threatening for the students.

## Adapting teamwork to individual needs

### Differentiated instruction and tailored support

Effective teamwork requires adapting teaching methods to meet students' diverse needs. Tomlinson [66] advocates differentiated instruction, which involves adjusting teaching approaches, materials and tasks to accommodate different learning needs and competency levels. Tailored support can significantly improve learning outcomes by ensuring that each student can engage with the material in a way that best suits their ability and background [67].

### Strategies for inclusivity and accommodating diverse student backgrounds

To create an inclusive learning environment, it is crucial to implement strategies that accommodate students' diverse backgrounds and needs. This includes:

1. Flexible grouping: allowing students to work in different team configurations based on their strengths and interests can enhance learning and ensure that all members feel valued and included. Tools, such as Belbin's role theory, can assist with flexible grouping and enhance team performance [68].
2. Cultural sensitivity: being aware of and responsive to the cultural backgrounds of students can help in designing team activities that are respectful and inclusive [69, 70].
3. Support for students with disabilities: providing appropriate accommodations, such as assistive technologies and adjusted timelines, ensures that students with disabilities can fully participate in teamwork [71, 72].

These strategies help create a supportive and equitable environment where all students can benefit from teamwork.

## Best practices for teamwork assessment

### Robust and transparent marking schemes

Effective assessment of teamwork relies on robust and transparent marking schemes as it is not always clear how individual skills within team‐based tasks are assessed [60]. Gibbs [73] emphasises the importance of clarity in assessment criteria to ensure fairness and to provide students with a clear understanding of what is expected. Transparent marking schemes help maintain consistency in evaluation and enhance student trust in the assessment process. Some tools have been developed to assist with assessing teamwork, but there is still a need for more robust and fair methods to reliably track the development of teamwork skills [74]. For this purpose, tools like the CATME‐BARS scale can be used [75], whilst Britton *et al*. [60] have described the development of a simplified version called Team Q for assessing individual development and contribution within team‐based assessments. These tools provide a practical and reliable way for instructors to assess teamwork, ensuring alignment between intended outcomes and the assessment.

### Assessing both process and product

Kennedy [76] suggests that assessing both the process and the final product of teamwork provides a more comprehensive evaluation. By evaluating the process, educators can recognise the efforts and contributions of individual members, which might not be evident in the final product alone [58]. This dual focus ensures that both the collaborative effort and the outcome are valued and rewarded [77].

To address the issue of unequal contribution, several techniques can be employed to individualise marks within team assessments:

1. Peer assessment: allowing team members to evaluate each other's contributions can help identify individual efforts and ensure fair distribution of marks [78].
2. Self‐assessment: encouraging students to reflect on their own contributions and learning can provide valuable insights and support fair assessment [79].
3. Weighted contributions: assigning different weights to individual contributions based on peer and self‐assessments can help in adjusting marks to reflect each member's efforts accurately [45, 46].

Implementing these best practices can enhance the fairness and effectiveness of teamwork assessment, ensuring that all students are recognised for their contributions and learning. Pragmatically, process‐driven assessment is also a strategy that is being proposed to help ensure academic integrity in the face of Generative AI [80].

## A framework for effective teamwork assessment

**Fig. 4:**
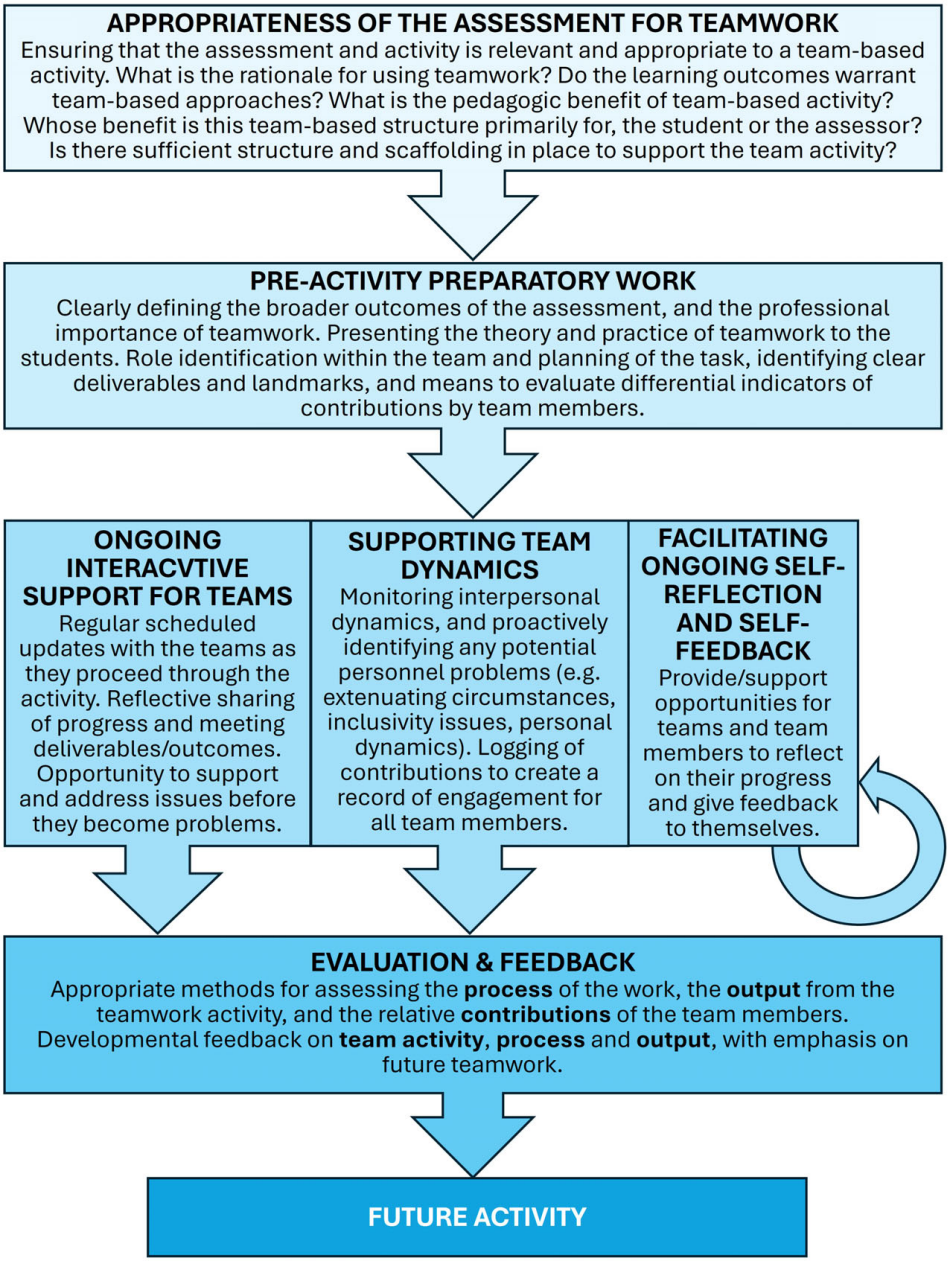
A suggested framework for managing a teamwork assignment. Each stage has key activities and considerations, leading to the development of teamwork skills and a positive impact on future team‐based activity.

Figure 4 suggests a framework for the management of an effective team‐based assignment. The model follows four key stages: (a) *Planning and rationale* (determining the purpose of the assessment and ensuring that a team‐based task is the most appropriate medium); (b) *Pre‐activity preparation* (supporting students in understanding the parameters of teamwork and how to manage a team‐based activity successfully); (c) *Support* (supporting student‐teams, managing the assignment process, and facilitating self‐reflection); (d) *Evaluation and feedback* (effective marking and peer evaluation approaches, and feedback for future development). Scaffolding the team activity in this way ensures that it is a supported learning activity for the students and enables the educator to head off any problems before they damage the cohesion of the team.

## Conclusion

This review highlights several critical strategies for enhancing the effectiveness of teamwork and its assessment in higher education. Structured approaches, including clear roles and responsibilities [33] and effective division of labour [32], are essential for promoting accountability and participation. Addressing common challenges like free‐riding and conflicts [40, 42] through strategies like team contracts and peer assessments ensures fairness and engagement. Furthermore, integrating active learning and real‐world applications [49, 50], along with technological tools [51], enhances student involvement and collaboration. Adapting team work to individual needs [66] and employing robust assessment techniques [73, 76] improve teamwork's effectiveness and equity.

### Future directions for research and practice in teamwork assessment

Future research should continue to explore innovative strategies for teamwork and assessment, focusing on longitudinal studies to understand long‐term impacts on student learning and professional readiness. Additionally, investigating the role of technology in facilitating remote and hybrid teamwork environments is increasingly relevant. Practitioners should emphasise ongoing training for educators in managing team dynamics and developing fair assessment criteria. By continuously refining these approaches, educators can better prepare students for collaborative professional environments, ensuring that teamwork remains a vital component of higher education.

## Conflict of interest

The authors declare no conflict of interest.

## Peer review

The peer review history for this article is available at https://www.webofscience.com/api/gateway/wos/peer‐review/10.1002/2211‐5463.13936.

## Author contributions

NF and SR conceived the initial project, all authors (NF, CP, ZP and SR) contributed equally to the literature searching and writing of the manuscript.

## References

[1] Francis NJ , Allen M and Thomas J (2022) Using Group Work for Assessment – An Academic's Perspective. Advance HE, Heslington.

[2] Mello LV , Varga‐Atkins T and Edwards SW (2021) A structured reflective process supports student awareness of employability skills development in a science placement module. FEBS Open Bio 11, 1524–1536. 10.1002/2211-5463.13158 PMC8167867 33835700

[3] Hamilton LG and Petty S (2023) Compassionate pedagogy for neurodiversity in higher education: a conceptual analysis. Front Psychol 14, 1093290. 36874864 10.3389/fpsyg.2023.1093290 PMC9978378

[4] De Prada E , Mareque M and Pino‐Juste M (2022) Teamwork skills in higher education: is university training contributing to their mastery? Psicol Reflex Crit 35, 5. 35141845 10.1186/s41155-022-00207-1 PMC8828815

[5] Zhang M (2023) “Groupwork or teamwork?”: creating the same page for improving team‐based learning experience. Commun Teach 37, 30–34.

[6] Goldsmith GR , Aiken ML , Camarillo‐Abad HM , Diki K , Gardner DL , Stipčić M and Espeleta JF (2024) Overcoming the barriers to teaching teamwork to undergraduates in STEM. CBE Life Sci Educ 23, es2. 38442149 10.1187/cbe.23-07-0128 PMC11235100

[7] Watson HR , Dolley M‐K , Perwaiz M , Saxelby J , Bertone G , Burr S , Collett T , Jeffery R and Zahra D (2022) ‘Everyone is trying to outcompete each other’: a qualitative study of medical student attitudes to a novel peer‐assessed undergraduate teamwork module. FEBS Open Bio 12, 900–912. 10.1002/2211-5463.13395 PMC9063444 35293162

[8] Telling K (2024) Why do students resist assessment by group‐work? Hearing critique in the complaint. Eur Educ Res J 23, 745–763.

[9] Francis NJ , Carss W , Cook S and Cowie B (n.d.) Yeah…Nah…Prioritising relationships or task content: student perspectives of group work. In preparation.

[10] Fittipaldi D (2020) Managing the dynamics of group projects in higher education: best practices suggested by empirical research. Univ J Educ Res 8, 1778–1796.

[11] Wilson L , Ho S and Brookes RH (2018) Student perceptions of teamwork within assessment tasks in undergraduate science degrees. Assess Eval High Educ 43, 786–799.

[12] Vygotsky LS (1978) Mind in Society Development of Higher Psychological Processes. Harvard University Press, Cambridge, MA.

[13] Mercer N (1995) The Guided Construction of Knowledge: Talk amongst Teachers and Learners. Multilingual Matters, Clevedon.

[14] Mercer N (1996) Words and Minds: How we Use Language to Think Together. Routledge, London.

[15] Mazur E (1996) Peer Instruction: A User's Manual. Prentice Hall, Upper Saddle River, NJ.

[16] Rutherford S (2015) E pluribus unum: the potential of collaborative learning to enhance microbiology teaching in higher education. FEMS Microbiol Lett 362, fnv191. 26459888 10.1093/femsle/fnv191

[17] Ellis R and Goodyear P (2009) Students' Experiences of e‐Learning in Higher Education. Routledge, New York, NY.

[18] Laurillard D (2012) Teaching as a Design Science: Building Pedagogical Patterns for Learning and Technology. Routledge, New York, NY.

[19] Jaques D (2000) Learning in Groups. 3rd edn. Routledge Farmer, New York, NY.

[20] QAA (2023) Subject Benchmark Statements.

[21] Scott JL , Moxham BJ and Rutherford SM (2014) Building an open academic environment – a new approach to empowering students in their learning of anatomy through ‘shadow modules’. J Anat 224, 286–295. 24117249 10.1111/joa.12112 PMC3931540

[22] Rutherford SM (2019) ‘Flying the Nest’: An Analysis of the Development of Self‐Regulated Learning during the Transition to Higher Education. University of Reading, Reading.

[23] Davis B (1993) Collaborative learning group work and study teams. In Tools for Teaching ( Davis BG , ed.), pp. 147–152. Jossey‐Bass, San Francisco, CA.

[24] Smith DP , Hoare A and Lacey MM (2018) Who goes where? The importance of peer groups on attainment and the student use of the lecture theatre teaching space. FEBS Open Bio 8, 1368–1378. 10.1002/2211-5463.12494 PMC6120247 30186739

[25] Lacey MM , Campbell SG , Shaw H and Smith DP (2020) Self‐selecting peer groups formed within the laboratory environment have a lasting effect on individual student attainment and working practices. FEBS Open Bio 10, 1194–1209. 10.1002/2211-5463.12902 PMC7327925 32438509

[26] Chang Y and Brickman P (2018) When group work Doesn't work: insights from students. CBE Life Sci Educ 17, ar52. 30183565 10.1187/cbe.17-09-0199 PMC6234829

[27] Noonan M (2013) The ethical considerations associated with group work assessments. Nurse Educ Today 33, 1422–1427. 23200886 10.1016/j.nedt.2012.11.006

[28] Hogan M and Young K (2021) Designing group assignments to develop Groupwork skills. J Inform Syst Educ 32, 274–282.

[29] Cortázar C , Nussbaum M , Alario‐Hoyos C , Goñi J and Alvares D (2022) The impacts of scaffolding socially shared regulation on teamwork in an online project‐based course. Internet High Educ 55, 100877.

[30] Race P (2019) The Lecturer's Toolkit: A Practical Guide to Assessment, Learning and Teaching. 5th edn. Routledge, New York, NY.

[31] Sublett LW , Johnston AM , Walther CAP , Seahorn C , Moreno GL and Brownlee L (2024) Speed‐interviewing for classroom group formation: how a clever twist on the classic “speed‐dating” tradition enhances small group coursework. Teach Psychol 51, 478–483.

[32] Sormunen E , Tanni M , Alamettälä T and Heinström J (2014) Students' group work strategies in source‐based writing assignments. J Assoc Inf Sci Technol 65, 1217–1231.

[33] Steiner S , Stromwall LK , Brzuzy S and Gerdes K (1999) Using cooperative learning strategies in social work education. J Soc Work Educ 35, 253–264.

[34] Bravo R , Catalán S and Pina JM (2019) Analysing teamwork in higher education: an empirical study on the antecedents and consequences of team cohesiveness. Stud High Educ 44, 1153–1165.

[35] Xu S (2024) Groupwork and collaborative learning: Chinese university students' struggles and strategies. High Educ Res Dev 43, 227–242.

[36] Dillenbourg P (1999) Chapter 1 (Introduction) What do you mean by ‘collaborative learning’? In Collaborative‐Learning: Cognitive and Computational Approaches ( Dillenbourg P , ed.), pp. 1–19. Elsevier, Oxford.

[37] Boud D and Bearman M (2024) The assessment challenge of social and collaborative learning in higher education. Educ Philos Theory 56, 459–468.

[38] Aronson E , Stephan C , Sikes J , Blaney N and Snapp M (1978) The Jigsaw Classroom. Sage Publication, Beverly Hills, CA.

[39] Magana AJ , Karabiyik T , Thomas P , Jaiswal A , Perera V and Dworkin J (2022) Teamwork facilitation and conflict resolution training in a HyFlex course during the COVID‐19 pandemic. J Eng Edu 111, 446–473. 37745167 10.1002/jee.20450 PMC9015229

[40] Payne B , Sumter M and Turner E (2005) Conflict resolution and group work. Acad Exch Q 9, 22–26.

[41] Nunkoo DK and Sungkur RK (2021) Team conflict dynamics & conflict management: derivation of a model for software organisations to enhance team performance and software quality. Glob Trans Proc 2, 545–552.

[42] Macfarlane B (2016) The performative turn in the assessment of student learning: a rights perspective. Teach High Educ 21, 839–853.

[43] Kerr NL and Bruun SE (1983) Dispensibility of member effort and group motivation losses; free rider effects. J Pers Soc Psychol 44, 78–94.

[44] Davies WM (2009) Groupwork as a form of assessment: common problems and recommended solutions. High Educ 58, 563–584.

[45] Gordon NA (2010) Group working and peer assessment—using WebPA to encourage student engagement and participation. Innov Teach Learn Inform Comput Sci 9, 20–31.

[46] Bates A (2019) Peer Evaluation of Groupwork with Canvas and Buddycheck on a Practical Life Sciences Module. University of Liverpool, Liverpool.

[47] Benning TM (2024) Reducing free‐riding in group projects in line with students' preferences: does it matter if there is more at stake? Act Learn High Educ 25, 242–257.

[48] Berkhout JJ , Helmich E , Teunissen PW , van der Vleuten CPM and Jaarsma ADC (2018) Context matters when striving to promote active and lifelong learning in medical education. Med Educ 52, 34–44. 28984375 10.1111/medu.13463

[49] Kriflik L and Mullan J (2007) Strategies to improve student reaction to group work. J Univ Teach Learn Pract 4, 17–32.

[50] Leight J , Barcelona RJ and Rockey DL (2010) Using collaborative learning technologies to facilitate effective group work. J Phys Educ Recreat Dance 81, 12–55.

[51] Moccozet L , Tardy C , Opprecht W and Léonard M (2013) Gamification‐Based Assessment of Group Work. Paper Presented at the 2013 International Conference on Interactive Collaborative Learning (ICL).

[52] Brar M , Douglas C and Lopez‐Capel E (2024) Using a gamification framework to increase student engagement with groupwork. Stud Engage High Educ J 5, 57–75.

[53] Redondo‐Rodríguez C , Becerra‐Mejías JA , Gil‐Fernández G and Rodríguez‐Velasco FJ (2022) Influence of gamification and cooperative work in peer, mixed and interdisciplinary teams on emotional intelligence, learning strategies and life goals that motivate university students to study. Int J Environ Res Public Health 20, 547. 36612869 10.3390/ijerph20010547 PMC9820013

[54] Toda AM , Klock ACT , Oliveira W , Palomino PT , Rodrigues L , Shi L , Bittencourt I , Gasparini I , Isotani S and Cristea AI (2019) Analysing gamification elements in educational environments using an existing gamification taxonomy. Smart Learn Environ 6, 16.

[55] Aldawi F and Maher A (2023) The role of Google docs in enhancing collaborative writing in higher education institutions. Paper Presented at the Proceeding – 2023 IEEE 3rd International Maghreb Meeting of the Conference on Sciences and Techniques of Automatic Control and Computer Engineering, MI‐STA 2023.

[56] Caple H and Bogle M (2013) Making group assessment transparent: what wikis can contribute to collaborative projects. Assess Eval High Educ 38, 198–210.

[57] Christie H and Morris N (2021) Using assessed blogs to enhance student engagement. Teach High Educ 26, 573–585.

[58] Mellor T (2012) Group work assessment: some key considerations in developing good practice. Plan Theory 25, 16–20.

[59] Johnson C , MacLeod MA and Visscher K (2022) The utility of a peer review application in interdisciplinary teamwork arrangements. Paper Presented at the in 50th Annual Conference of the European Society for Engineering Education, SEFI 2022.

[60] Britton E , Simper N , Leger A and Stephenson J (2017) Assessing teamwork in undergraduate education: a measurement tool to evaluate individual teamwork skills. Assess Eval High Educ 42, 378–397.

[61] Bacon DR , Stewart KA and Silver WS (2019) Republication of “lessons from the best and worst student team experiences: how a teacher can make the difference”. J Manage Educ 43, 550–572.

[62] Chapman KJ and van Auken S (2001) Creating positive group project experiences: an examination of the role of the instructor on Students' perceptions of group projects. J Mark Educ 23, 117–127.

[63] Beeson H and Byles R (2020) Creative solutions to common groupwork problems. J Learn Dev High Educ doi: 10.47408/jldhe.vi19.622

[64] Veine S , Anderson MK , Andersen NH , Espenes TC , Søyland TB , Wallin P and Reams J (2020) Reflection as a core student learning activity in higher education – insights from nearly two decades of academic development. Int J Acad Dev 25, 147–161.

[65] Rutherford SM and Prytherch ZC (2016) Assessment ‘for’ Learning: Embedding Digital Literacy and Peer‐Support of Learning into an Assessment. In Handbook of Research on Engaging Digital Natives in Higher Education Settings ( Pinheiro MM and Simões D , eds), pp. 121–153. IGI Global, Hershey, PA.

[66] Tomlinson CA (2001) How to Differentiate Instruction in Mixed‐Ability Classrooms. Pearson Education, Upper Saddle River, NJ.

[67] Ireh M and Ibeneme OT (2011) Differentiating instruction to meet the needs of diverse technical/technology education students at the secondary school level. Afr J Teach Educ 1, doi: 10.21083/ajote.v1i1.1581

[68] Aranzabal A , Epelde E and Artetxe M (2022) Team formation on the basis of Belbin's roles to enhance students' performance in project based learning. Educ Chem Eng 38, 22–37.

[69] Popov V , Brinkman D , Biemans HJA , Mulder M , Kuznetsov A and Noroozi O (2012) Multicultural student group work in higher education: an explorative case study on challenges as perceived by students. Int J Intercult Relat 36, 302–317.

[70] Eden C , Chisom O and Adeniyi I (2024) Cultural competence in education: Strateguies for fostering inclusivity and diversity awareness. Int J Appl Res Soc Sci 6, 383–392.

[71] Majoko T (2018) Participation in higher education: voices of students with disabilities. Cogent Educ 5, 1542761.

[72] Aguirre A , Carballo R and Lopez‐Gavira R (2021) Improving the academic experience of students with disabilities in higher education: faculty members of social sciences and law speak out. Innovations 34, 305–320.

[73] Gibbs G (2009) The Assessment of Group Work: Lessons from the Literature. Assessment Standards. Knowledge Exchange, Oxford.

[74] Parker R , Hodierne L , Anderson ES , Davies RS and Elloy M (2019) Academic ability and teamworking in medical students. Clin Teach 16, 209–213. 29806734 10.1111/tct.12800

[75] Baviera T , Baviera‐Puig A and Escribá‐Pérez C (2022) Assessing team member effectiveness among higher education students using 180° perspective. Int J Manage Educ 20, 100702.

[76] Kennedy D (2006) Writing and Using Learning Outcomes: A Practical Guide. University College Cork, Cork.

[77] Gledhill M and Smith P (1996) Student Perceptions of Learning with Reference to Group Work. Staff Development Unit, Buckinghamshire College of Higher Education, Buckinghamshire.

[78] Sridharan B , McKay J and Boud D (2023) The four pillars of peer assessment for collaborative teamwork in higher education. In The Power of Peer Learning: Fostering Students' Learning Processes and Outcomes ( Noroozi O and De Wever B , eds), pp. 3–24. Springer International Publishing, Cham.

[79] Planas‐Lladó A , Feliu L , Arbat G , Pujol J , Suñol JJ , Castro F and Martí C (2021) An analysis of teamwork based on self and peer evaluation in higher education. Assess Eval High Educ 46, 191–207.

[80] Smith DP and Francis NJ (2024) Process not product in the written assessment. In Using Generative AI Effectively in Higher Education ( Beckingham S , Lawrence J , Powell S and Hartley P , eds), pp. 115–126. Routledge, London.

